# Ultradispersed Nanoarchitecture of LiV_3_O_8_ Nanoparticle/Reduced Graphene Oxide with High-Capacity and Long-Life Lithium-Ion Battery Cathodes

**DOI:** 10.1038/srep19843

**Published:** 2016-01-28

**Authors:** Runwei Mo, Ying Du, David Rooney, Guqiao Ding, Kening Sun

**Affiliations:** 1 Academy of Fundamental and Interdisciplinary Sciences, Harbin Institute of Technology, Harbin 150001, (China); 2 State Key Laboratory of Functional Materials for Informatics, Shanghai Institute of Microsystem and Information Technology, Shanghai, 20050, (China); 3 School of Chemistry and Chemical Engineering, Queen's University Belfast, Belfast, BT9 5AG, (Northern Ireland)

## Abstract

Lack of high-performance cathode materials has become the major barriers to lithium-ion battery applications in advanced communication equipment and electric vehicles. In this paper, we report a versatile interfacial reaction strategy, which is based on the idea of space confinement, for the synthesis of ultradispersed LiV_3_O_8_ nanoparticles (~10 nm) on graphene (denoted as LVO NPs-GNs) with an unprecedented degree of control on the separation and manipulation of the nucleation, growth, anchoring, and crystallization of nanoparticles in a water-in-oil emulsion system over free growth in solution. The prepared LVO NPs-GNs composites displayed high performance as an cathode material for lithium-ion battery, including high reversible lithium storage capacity (237 mA h g^−1^ after 200 cycles), high Coulombic efficiency (about 98%), excellent cycling stability and high rate capability (as high as 176 mA h g^−1^ at 0.9 A g^−1^, 128 mA h g^−1^ at 1.5 A g^−1^, 91 mA h g^−1^ at 3 A g^−1^ and 59 mA h g^−1^ at 6 A g^−1^, respectively). Very significantly, the preparation method employed can be easily adapted and may opens the door to complex hybrid materials design and engineering with graphene for advanced energy storage.

With the advantages of high energy density, long lifespan and environmental benignity, lithium-ion batteries (LIBs) have become the predominant power source for applications in electric vehicles (EVs), and renewable energy storage in smart grids^1^,^2^,^3^,^4^,^5^,^6^,^7^,^8^,^9^,^10^,^11^. Lack of high-performance cathode materials has become a technological bottleneck for the commercial development of advanced LIBs^12^. Lithium trivanadate (LiV_3_O_8_) as cathode materials have drawn remarkable attention because of its high specific energy, good rate capacity and long-term stability, etc^13^,^14^,^15^,^16^. Based on its theoretical calculation, around 3Li^+^ (ca. 280 mAh g^−1^) can be reversibly insertion/deinsertion upon the crystallized LiV_3_O_8_ electrode. However, it was also reported that for amorphous one, a maximum amount of 4.5Li^+^ (ca. 419 mAh g^−1^) could be demonstrated^17^, explained by that for the amorphous LiV_3_O_8_, more organized arrangements at the subunit level facilitate the inclusion of more lithium ions per unit cell^18^. However, many potential electrode materials (e.g., LiV_3_O_8_) in lithium-ion batteries are limited by poor electron transport, slow Li-ion diffusion in electrodes, and suffered from fragile surface properties at the interface of electrode/electrolyte at high charge-discharge rates^19^,^20^,^21^. To address these issues, various strategies have been developed to improve the structural integrity and electrical conductivity of LiV_3_O_8_-based materials, such as optimizing particle size^22^,^23^ or morphology^24^,^25^,^26^,^27^. Although nanostructuring has been successful in extending the cycle life of silicon, nanostructured electrodes have introduced new fundamental challenges, including higher surface area, low tap density and generally poor electrical properties due to the higher interparticle resistance. The high surface area increases side reactions with the electrolyte and lowers the coulombic efficiency. Finally, electrical contact between the nanoparticles is easily altered or diminished by lithiation/delithiation processes during cycling, severely decreasing the cycle life of the electrode. To the best of our knowledge, stable cycling (200 cycles) with reversible capacity higher than 200 mA h g^−1^ have rarely been reported for LiV_3_O_8_ cathodes.

Graphene (G), a monolayer of carbon atoms arranged in a honeycomb network, is one of the most favorable carbonaceous materials for constructing functional composites and/or hybrids because of its unique 2D nanostructure, excellent electron conductivity, good chemical stability, and very high theoretical surface area (2600 m^2^ g^−1^)^28^,^29^,^30^,^31^,^32^,^33^. Recently, graphene has shown great potential in LIBs applications^34^,^35^. Several methods, including the direct deposition process and a two-step approach, have been extensively used to form oxide nanoparticles on chemically derived graphene sheets^36^,^37^,^38^. However, one of the challenges associated with the integration is how to ensure the nucleation and growth of nanoparticles, selectively on graphene sheets over free growth in solution because it is difficult to match their compatibilities and interactions in addition to regulating the reduction of graphene oxide (GO)^39^. Another challenge is how to control the particle size, uniform dispersion, and strong coupling, as well as keeping the reduced graphene oxide (rGO) sheets individually separated^40^. It is noteworthy, that the small particle size, uniform dispersion, and strong coupling with the graphene sheets are crucial factors for improving cell performance^41^,^42^,^43^ because small particle size plus good dispersion can endow the composite electrode a superior high surface area to improve the compact contact of active materials/supports are favorable for such activation processes, but it could also bring the required conductivity to individual nanoparticles and shorten the diffusion length for Li ions, which are beneficial for high lithium storage and rate capability, respectively. However, it remains a big challenge to grow small nanoparticle (~10 nm), uniform dispersion, and strong coupling with the graphene sheets over free growth in solution for materials with sophisticated compositions, such as LiV_3_O_8_, which is a promising cathode materials for LIBs.

Herein, we report a versatile interfacial reaction strategy, which is based on the idea of space confinement, for the synthesis of ultradispersed LiV_3_O_8_ nanoparticles (~10 nm) and strong coupling on graphene nanosheets (denoted as LVO NPs-GNs) with an unprecedented degree of control on the precise separation and manipulation of nanoparticles nucleation, growth, anchoring, and crystallization on graphene oxide (GO) in a water-in-oil emulsion system over free growth in solution. For comparison LiV_3_O_8_ particles were freely grown on graphene sheets in solution via a traditional sol-gel method (denoted as SG-LVO-GNs). Such LVO NPs-GNs composites with the small particle size and uniform distribution of LVO NPs could be controlled with good reproducibility, and anchoring firmly on the GNs is advantageous for inhibiting aggregation and offers a direct short pathway for Li^+^ diffusion, which are beneficial for high capacity and rate capability. Specifically, this well LVO NPs-GNs composites is able to deliver high reversible capacities with superlative cyclic capacity retention at different current rates for prolonged cycling, and exhibit excellent high-rate performance at a current density as high as 6 A g^−1^ as an cathode material for LIBs. Very significantly, this facile method may offer an attractive alternative approach for preparation of the graphene-based composites as high-performance electrodes for lithium-ion batteries.

## Results

**Figure 1:**
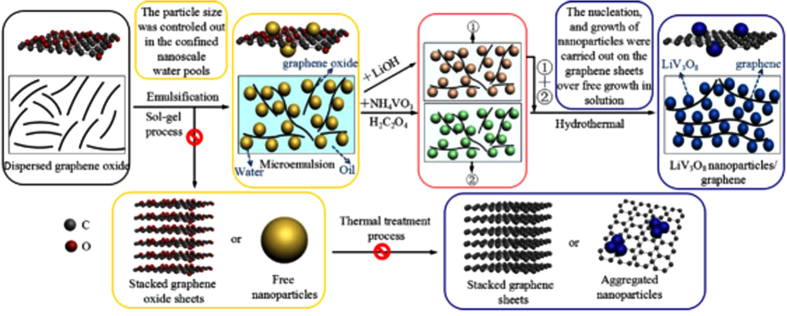
Schematic diagram of synthesis steps for LVO NPs-GNs composites.

In this versatile interfacial reaction design strategy, the unprecedented control of the hybrid materials is achieved by the rational separation and precise manipulation of the synthesis processes (involving LVO NPs nucleation, growth, anchoring, crystallization on GO in the confined nanoscale water pools) (Fig. 1). Firstly, as-prepared graphite oxide was dispersed in water by ultrasonication for 40 min, followed by a low-speed centrifugation to get rid of any un-dispersed GO nanosheets (Figure S1, ESI†). The second step was the formation of a water-in-oil emulsion containing GO. The basal plane of GO is partially hydrophobic while the functional groups attached on their surface make them hydrophilic. The flexible GO could restack around water droplets with using CTAB as sufactant. The third step was the synthesis of uniformly nonaggregated LVO NPs. All the stages of nucleation, growth, and crystallization of LVO NPs were carried out in the confined nanoscale water pools of the W/O microemulsions. Fourthly, the hydrothermal treatment of the microemulsions is an effective route for the crystallization of LVO NPs under mild conditions. Simultaneously, GO was reduced to mildly oxidized graphene oxide sheets (rmGO) under the hydrothermal condition. Finally, the mixture was annealed at 400 °C for 2 h under nitrogen flow to remove the residual water molecules and functional groups in the rmGO, and to improve the crystallinity of LVO NPs in the obtained composites, which would further improve its electrochemical performance.

**Figure 2:**
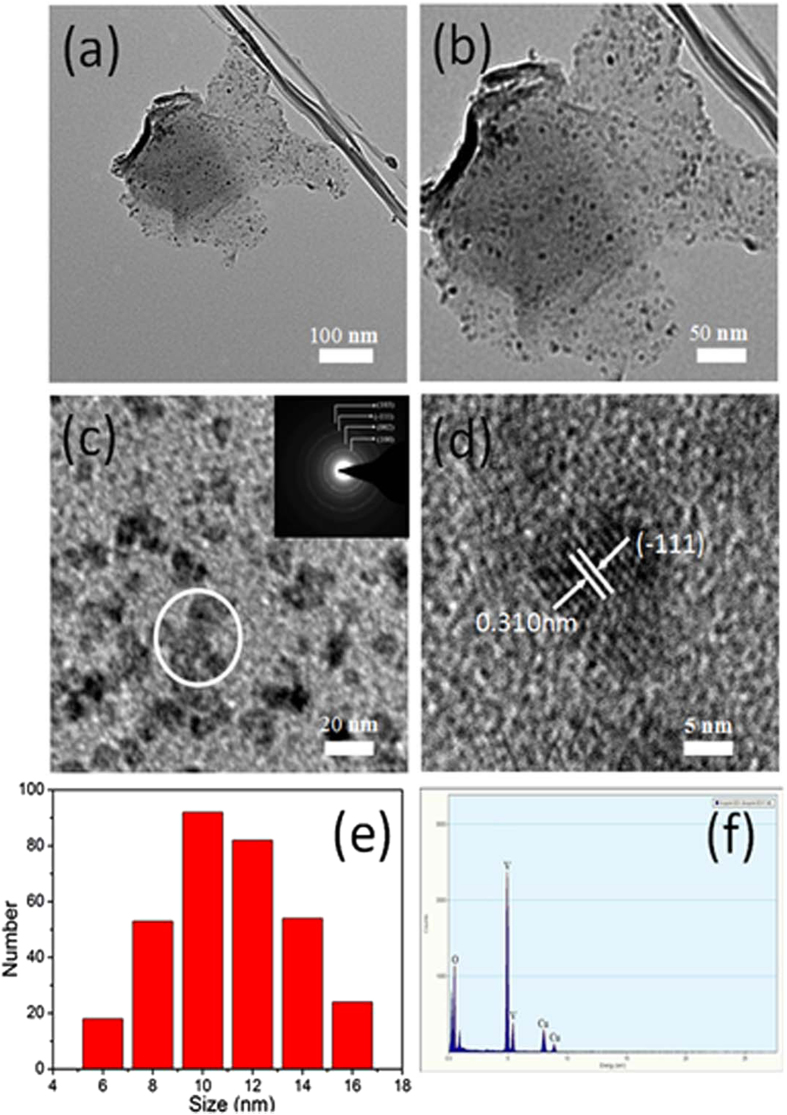
(**a–c**) TEM images and (**d**) high-resolution TEM image of the LVO NPs-GNs composites. Inset in (**c**) is the electronic diffraction pattern corresponding to the LVO NPs. (**e**) Histogram diagram of the LVO diameter. (**f**) EDX spectrum of the LVO NPs-GNs composites. The copper signals are from the Cu grids.

The size and morphology of the as-prepared products were characterized by TEM. The low-resolution TEM images (Fig. 2a,b) of an individual composited nanosheet exhibit a curved characteristic and a low contrast, revealing an ultrathin thickness of the nanosheets with LVO NPs loading. The high dispersion and uniform coverage on the GNs of the nanoparticles are clear under enlarged TEM characterization (Fig. 2c). The selected area electronic diffraction pattern (inset in Fig. 2c) corresponding to LVO NPs-GNs gives a set of diffraction rings, which can be clearly assigned to the diffractions of the {100}, {002}, {−111}, and {103} planes, respectively, of the layered-type structure of LiV_3_O_8_. In addition, High-resolution TEM showed the crystal lattice fringes throughout the entire LVO NPs formed on graphene (Fig. 2d), demonstrating the well crystalline nature of the nanocrystals with a regular spacing of 0.310 nm, which is in good agreement with the (−111) plane of LiV_3_O_8_. Moreover, the size distribution of LVO NPs on GNs was determined by measuring the NP diameters in TEM images, as shown in Fig. 1e. The size distribution curve of the LVO NP displays a mean diameter centered at ~10 nm with a standard deviation of about 10%, which is consistent with that of pure LVO (Figure S2, ESI†). The EDX analysis shows that the LVO NPs-GNs composites are composed of O, V, and Cu. The Cu peaks are from the copper grid where the TEM sample is placed on. Therefore, the homogeneous and ultradispersed nature of these small LiV_3_O_8_ nanoparticles on graphene can be confirmed by the scanning TEM image. However, it is worth noting that the traditional sol-gel strategy for the synthesis of LVO NPs on graphene (SG-LVO-GNs) composites shows the large aggregated nanoparticles and restacked graphene sheets (Figure S3, ESI†). It also demonstrates that the LVO NPs-GNs can effectively prevent nanoparticle aggregation and graphene nanosheet restacking in a water-in-oil emulsion system.

**Figure 3:**
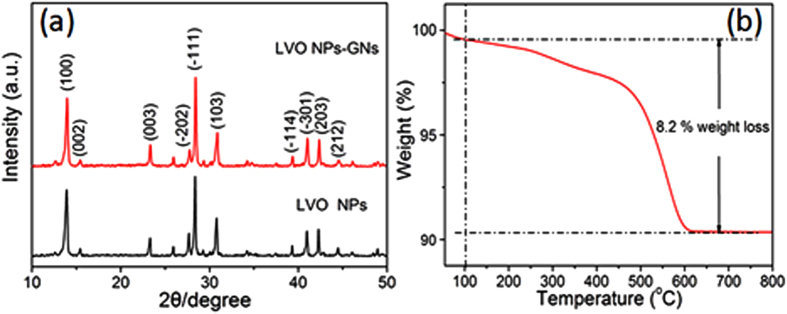
(**a**) X-Ray diffraction (XRD) patterns of LVO NPs, and LVO NPs-GNs. (**b**) Thermogravimetric analysis (TGA) of the LVO NPs-GNs.

Powder X-ray diffraction (XRD) experiments were carried out to reveal the crystallographic phases of the final product, with the result shown in Fig. 3a. All distinct XRD peaks from the sample of LVO NPs-GNs are well indexed to the layered-type structure of LiV_3_O_8_ with the P_21/m_ space group (JCPDS 72-1193). Diffraction peaks which might appear for graphene are absent, most likely because they are below the limits of detection by XRD or, in the case of the graphene (002) peak, because it is likely to be eclipsed by the LiV_3_O_8_ (003) peak. The absence of a reflection peak at approximately 11° indicates that the GO cannot be distinguished, consistent with full or partial reduction to graphene. Raman spectroscopy is a non-destructive approach to characterize graphitic materials, in particular to determine the ordered and/or disordered crystal structure of graphene sheets^44^. In the Raman spectrum, the D peak usually corresponds with the k-point phonons of A1g symmetry while the G peak is related to the E_2g_ phonons of C_sp2_ atoms^45^, and their relative intensity gives the clue to the ordered and/or disordered crystal structures of graphene. The Raman spectra of the obtained LVO NPs-GNs composites and GNs are shown in Figure S4 (ESI†), a broad D band (1330 cm^−1^) and a broad G band (1590 cm^−1^) are observed in both samples^46^. It is well known that the intensity ratio of the D to G band (I_D_/I_G_) reflects the graphitization degree of carbonaceous materials and the defect density^47^. The I_D_/I_G_ for LVO NPs-GNs (1.37) is much larger than that for GNs (1.26), showing the transformation of graphene sheets from graphene oxide sheets after the thermal treatment. The intensity of the characteristic peak of the D band is slightly stronger than that of the G band, indicating the existence of graphene in the composites. Thermogravimetric analysis (TGA) was employed to determine the amount of graphene in the LVO NPs-GNs (Fig. 3b). It is immediately apparent that a significant weight loss takes place at 500–600 °C, and the weight fraction of the graphene support is measured to be ~8.2%. In addition, According to Brunauer-Emmett-Teller (BET) analysis, a total specific surface area of 112.8 m^2^ g^−1^ is obtained (Figure S5, ESI†). Such high surface area provides more surface-active sites and makes the diffusion of the liquid electrolyte into the electrodes more easily, leading to an enhancement of the electrical performance.

**Figure 4:**
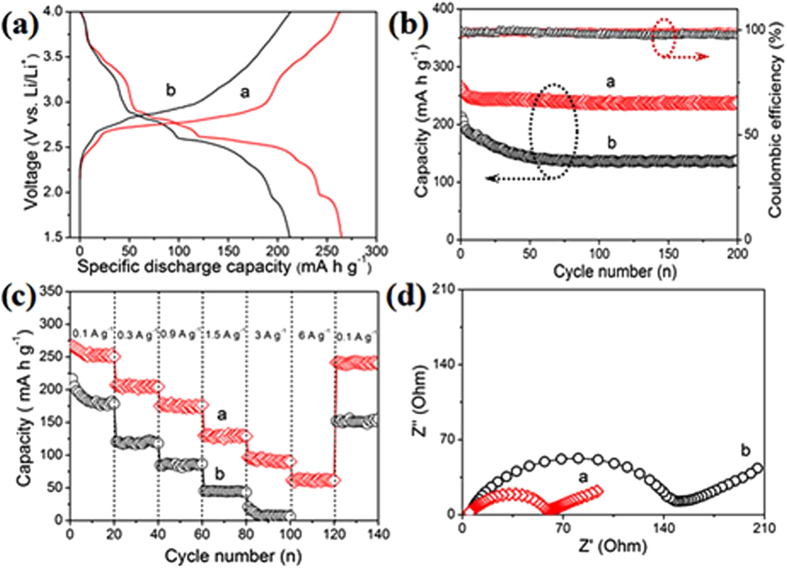
(**a**) First-cycle discharge curves of the (**a**) LVO NPs-GNs and (**b**) LVO NPs electrodes. (**b**) Left axis: Cycling performance of the electrodes: (**a**) LVO NPs-GNs and (**b**) LVO NPs electrodes. The electrodes were charged-discharged between 1.5 and 4 V (vs. Li/Li^+^) at current densities of 0.1 A g^−1^ (1C = 0.3 A g^−1^); Right axis: Coulombic efficiency of the LVO NPs-GNs electrode. (**c**) Rate-performance of the electrodes: (**a**) LVO NPs-GNs and (**b**) LVO NPs. (**d**) Nyquist plots of the electrodes of (**a**) LVO NPs-GNs and (**b**) LVO NPs.

The lithium-insertion/extraction properties of the LVO NPs-GNs as an cathode material for LIBs were investigated by galvanostatic charge/discharge measurements over a voltage range of 1.5–4.0 V. Figure 4a shows first specific discharge capacity of as high as 264 mA h g^−1^ observed on LVO NCs-GNs at 0.1 A g^−1^ based on the mass of the active material LVO, which is much higher than that of LVO NCs (212 mA h g^−1^). This is considered to be because the ultrathin graphene nanosheets act as conducting routes between the LVO NPs, resulting in significantly reduced contact resistance. Remarkably, the reversible capacity of LVO NPs-GNs is still stable at 237 mA h g^−1^ even after 200 cycles, while the reversible capacities of LVO NPs gradually reduce to 138 mA h g^−1^ (Fig. 4b). Note that the coulombic efficiencies of LVO NPs-GNs are about 98% during cycling at current densities of 0.3 A g^−1^ as shown in Fig. 4b. As a sum result, the much better cyclability of LVO NPs-GNs composite is attributed to the following several merits. First, the small size of LVO NPs could significantly reduce the strain generated during the lithiation/delithiation processes and then suppress the fracture of LVO NPs. Second, on account of the homogeneous dispersion of LVO NPs, the generated stress upon cycling would evenly distribute in the whole composite as well as the electrode, preventing local cracking. Finally, the interconnected graphene network structure not only prevents particle aggregation, but also maintains the structural integrity and electrical conductivity of the electrode.

More exciting results come from the rate performance of LVO NPs-GNs in the Fig. 4c. It is obvious that LVO NPs-GNs have much better electrochemical performance than that of pure LVO NPs. As demonstrated in Fig. 4c, the reversible capacity of LVO NPs-GNs is stable at 251 mA h g^−1^ after 20 cycles at a rate of 0.1 A g^−1^. Upon increasing the discharge-charge rates to 0.3, 0.9, 1.5 and 3 A g^−1^, its reversible capacities are maintained at 204, 176, 128 and 91 mA h g^−1^, respectively. However, the reversible capacity of LVO NPs is stable at 178 mA h g^−1^ after 20 cycles at a rate of 0.1 A g^−1^. Upon increasing the discharge-charge rates to 0.3, 0.9, 1.5 and 3 A g^−1^, its reversible capacities are maintained at 117, 86, 55 and 25 mA h g^−1^, respectively. It’s worth noting that the specific capacity of LVO NPs-GNs at the rate of 6 A g^−1^ is as high as 59 mA h g^−1^, 8 times higher than that of the LVO NPs without graphene support. The above results demonstrate that the obtained LVO NPs-GNs show superior rate capability. The superior rate capability of LVO NPs-GNs structure should benefit from the combination of the high electric conductivity offered by graphene support and the short diffusion path for both electrons and ions provided by the small LVO particles and the graphene network structure. In addition, Li^+^ diffusivity should be lower in more aggregated particles, and therefore, LVO NPs size distribution is strongly related to battery cycling performance and rate capability. Importantly, the cell capacity could recover to the original values when the current density returns to lower current density after high rate cycling, indicating that the unique structure of LVO NPs-GNs composite could preserve the integrity of the electrode and thus tolerant to varied charge and discharge currents, which is important for high power applications of rechargeable batteries. To further understand the outstanding electrochemical performance of LVO NPs-GNs composite, electrochemical impedance spectroscopy (EIS) measurements (Fig. 4d) were carried out for LVO NPs and LVO NPs-GNs electrodes after the 10th cycle at a rate of 0.1 mA g^−1^. Apparently, the LVO NPs-GNs electrode shows a much lower resistance than the pure LVO NPs electrode (66 vs. 142 Ω). Furthermore, the interconnected graphene network structure provides a conductive network for electronic transport from LVO NPs within the whole electrode and thus decreases resistance.

**Figure 5:**
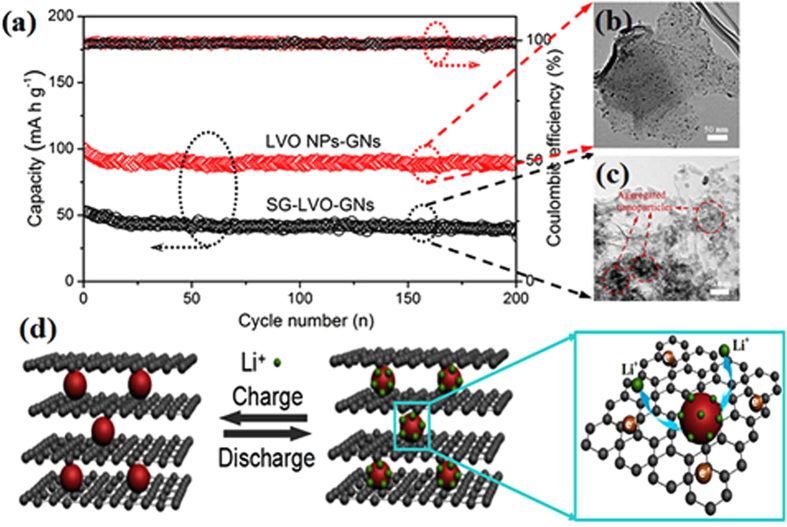
(**a**) Left axis: cycling performance of the LVO NPs-GNs and SG-LVO-GNs electrodes at current densities of 3 A g^−1^. Right axis: coulombic efficiency of the LVO NPs-GNs and SG-LVO-GNs electrodes at current densities of 3 A g^−1^. (**b**,**c**) TEM images of LVO NPs-GNs and SG-LVO-GNs. (**d**) Schematic drawing of the charge/discharge processes of the LVO NPs-GNs electrode cathode.

To investigate crucial factors for improving cell performance, we determined the high-rate cycling performance of the LVO NPs-GNs and SG-LVO-GNs composites under identical test conditions as shown in Fig. 5a. The cycling performance of the LVO NPs-GNs and SG-LVO-GNs composites are then evaluated by charge/discharge at current densities of 3 A g^−1^. After 200 cycles, it is clear that the SG-LVO-GNs composites have a lower capacity than that of the LVO NPs-GNs composites. That is because the large aggregated nanoparticles and restacked graphene sheets are produced in solution via a sol-gel method, which leads to lower active sites for lithium storage (Fig. 5c)^44^,^48^. The higher aggregation characteristics via a sol-gel method leads to the difficulty of Li^+^ mobility into the core side of the active material during the discharge. As a result, the nanoparticle aggregation has a negative influence on the electrochemical performances including the capacity, charge/discharge cycling performance, and the rate capability. It also demonstrates that the small particle size, uniform dispersion, and strong coupling with the graphene sheets are crucial factors for improving cell performance (Fig. 5b). Additionally, after the high-current-density measurements, the morphology of LVO NPs-GNs is still well retained (Figure S6, ESI†). This means that the well connections between nanoparticles and graphene sheets are responsible for the good cycling performance during lithium-insertion/extraction processes.

## Discussion

Consequently, the high capacity, remarkable rate capability, and outstanding cycle stability of the LVO NPs-GNs may be caused by the synergistic coupling effects in the LVO NPs-GNs composites (Fig. 5b)^49^,^50^: (i) The small particle size and uniform distribution of LiV_3_O_8_ nanoparticles that reduces the path lengths for Li^+^ and electron transfer between active particles, significantly increasing the electroactive zone^41^; (ii) the ultrathin graphene nanosheets not only can act as a conductive substrate to improve the electron transport, offer a direct short pathway for Li^+^ diffusion and significantly reduced contact resistance, but also restrains the aggregation of nanoparticles and maintains the nanostructure during long cycles^48^; (iii) the unique nanostructure not only provides a high surface area for interfacial lithium storage but also maintains the integrity of the electrode during the charge and discharge processes^51^, which is responsible for the high rate capability and cycling stability.

In summary, we describe a versatile interfacial reaction strategy, which is based on the idea of space confinement, for the synthesis of LVO NPs-GNs (~10 nm) composites in a W/O emulsion system. It is demonstrated that as the cathode material of LIBs, LVO NPs-GNs composite with the combination of small particle size, uniform dispersion and strong coupling with the graphene sheets render long cycling stability and high rate capability. At a current density of 0.1 A g^−1^, a capacity of 237 mAh g^−1^ was maintained after 200 cycles. Even at much higher current density of 6 A g^−1^, a reversible capacity of 55 mAh g^−1^ was obtained. All of these results demonstrate that the as-prepared LVO NPs-GNs composite encompasses three key advantages of small particle size, uniform dispersion, and strong synergistic coupling effects. Importantly, these results would shed light on the great potential application of cathode materials as high capacity electrode with high rate capability for next generation LIBs. Very significantly, this work opens the door to the rational design and controllable synthesis of useful graphene-based materials for advanced energy storage.

## Methods

### Synthesis of graphene oxide (GO)

GO was prepared from graphite flakes by a modified Hummers method^52^,^53^. 1.0 g of graphite flakes, 1.0 g of NaNO_3_ and 46 mL of concentrated H_2_SO_4_ were mixed together in an ice bath for 4 h. Then 6.0 g of KMnO_4_ was added slowly into the solution. Afterwards, the ice bath was removed and the suspension was stirred for another 3 days. Then the suspension was further treated with 200 mL of warm water (~60 °C) and 10 mL of H_2_O_2_ (30%). The mixture was centrifuged at 4000 rpm and washed with diluted HCl and water to neutral. Finally, a homogeneous GO aqueous dispersion (1 mg mL^−1^) was obtained for further use.

### Preparation of LVO NCs-GNs composites

In a typical experimental procedure, 5.6 g of Cetyltrimethyl Ammonium Bromide (CTAB) was dissolved in a mixture of 10 ml of n-pentanol and 60 ml of n-hexane. Then, 10 ml of GO aqueous dispersion (1 mg mL^−1^) was slowly poured and intensely stirred for 20 min at room temperature resulting in the formation of a golden water-in-oil emulsion. Then 1.8 mmol of NH_4_VO_3_ and 2 mmol of H_2_C_2_O_4_ or 1 mmol of LiOH•H_2_O was added to this golden water-in-oil emulsion while stirring. After 10 min of vigorous agitation, equivalent volumes of two separate microemulsion solutions containing NH_4_VO_3_ and H_2_C_2_O_4_ or LiOH•H_2_O were mixed rapidly. The suspension of the microemulsion were transferred into stainless steel autoclaves and heated at 200 °C for 16 h, followed by cooling to room temperature naturally. After cooled to ambient temperature, the black participates was obtained by centrifugation, and then washed for several times with distilled water. After drying in the vacuum oven at 70 °C overnight, as-synthesized LiV_3_O_8_-graphene was annealed at 400 °C for 2 h in an nitrogen flow in order to remove the residual water molecules and functional groups in the rGO, and to improve the crystallinity of LiV_3_O_8_ in the obtained composites, which would further improve its electrochemical performance. For comparison, Pure LiV_3_O_8_ nanoparticles were also prepared by the similar procedure without any GNs.

### Preparation of SG-LVO-GNs composites

In a typical sol-gel experimental procedure, 10 ml of GO aqueous dispersion (1 mg mL^−1^) was slowly poured and intensely stirred for 20 min at room temperature resulting in the formation of a golden transparent solution. Then 1.8 mmol of NH_4_VO_3_ and 2 mmol of H_2_C_2_O_4_ or 1 mmol of LiOH•H_2_O was added to this golden transparent solution while stirring. After 10 min of vigorous agitation, equivalent volumes of two separate solutions containing NH_4_VO_3_ and H_2_C_2_O_4_ or LiOH•H_2_O were mixed rapidly. The suspension of the solution were transferred into stainless steel autoclaves and heated at 200 °C for 16 h, followed by cooling to room temperature naturally. After cooled to ambient temperature, the black participates was obtained by centrifugation, and then washed for several times with distilled water. After drying in the vacuum oven at 70 °C overnight, as-synthesized LiV_3_O_8_-graphene was annealed at 400 °C for 2 h in an nitrogen flow in order to remove the residual water molecules and functional groups in the rGO, and to improve the crystallinity of LiV_3_O_8_ in the obtained composites, which would further improve its electrochemical performance.

### Characterizations

The resulting sample was characterized by means of X-ray diffraction (XRD, Rigaku D/max-B) with monochromated Cu K radiation at a scanning rate of 2° min^−1^ in the range of 10–50°. Morphology of products was characterized by scanning electron microscopy (SEM, Hitachi S4800) and high resolution transmission electron microscopy (HRTEM, JEM-2100) with an accelerating voltage of 200 KV. TG analysis was carried out with a TG/DTA6200 instrument. The specific surface area was measured by the Brunauer-Emmett-Teller (BET) method using ASAP2020. Additionally, Raman spectra were recorded at room temperature by employing an InVia Raman spectrometer using 633 nm red laser with 10% intensity to determine the extent of graphitic disorder.

### Battery assemble process and Electrochemical measurements

The LVO NPs-GNs active material powder (~240 mg) was mixed with Super P carbon black and polyvinyldifluoride (PVDF, Kynar HSV 900), with weight ratio of 80:10:10, in N-Methylpyrrolidone (NMP) solvent to produce an electrode slurry. The mass of active materials was calculated at ca. 3.2 mg and integrated into two-electrode CR2025-type coin cells for electrochemical measurements, with LVO NPs-GNs electrode as cathode, metallic lithium foil as anode, porous polypropylene film as separator; electrolyte was 1.0 M LiPF_6_ dissolved in ethylene carbonate (EC) and dimethyl carbonate (DMC) and diethyl carbonate (DEC) at a volumetric ratio of 1:1:1. The discharge-charge tests were conducted at various rates within a voltage window from 1.5 V to 4.0 V (vs. Li^+^/Li) on the Battery Testing System (BTS) battery testing system (Neware, Shenzhen, China). Electrical impedance spectroscopy (EIS) experiments were carried out on a Parstat 2273 advanced electrochemical systems in the frequency range mainly from 100 kHz to 10 mHz with the a.c. signal amplitude of 5 mV.

## Additional Information

**How to cite this article**: Mo, R. *et al*. Ultradispersed Nanoarchitecture of LiV_3_O_8_ Nanoparticle/Reduced Graphene Oxide with High-Capacity and Long-Life Lithium-Ion Battery Cathodes. *Sci. Rep*. **6**, 19843; doi: 10.1038/srep19843 (2016).

## Supplementary Material

Supplementary Information

## References

[b1] Maier J. Nanoionics: Ion Transport and Electrochemical Storage in Confined Systems. Nat. Mater 4, 805–815 (2005). 16379070 10.1038/nmat1513

[b2] Dunn B., Kamath H. & Tarascon J. -M. Electrical Energy Storage for the Grid: A Battery of Choices. Science 334, 928–935 (2011). 22096188 10.1126/science.1212741

[b3] Kang K. S., Meng Y. S., Breger J., Grey C. P. & Ceder G. Electrodes with High Power and High Capacity for Rechargeable Lithium Batteries. Science 311, 977–980 (2006). 16484487 10.1126/science.1122152

[b4] Kang B. & Ceder G. Battery Materials for Ultrafast Charging and Discharging. Nature 458, 190–193 (2009). 19279634 10.1038/nature07853

[b5] Ji X., Lee K. T. & . & Nazar L. F. A Highly Ordered Nanostructured Carbon-Sulphur Cathode for Lithium-Sulphur Batteries. Nat. Mater 8, 500–506 (2009). 19448613 10.1038/nmat2460

[b6] Guo Y.-G., Hu J.-S. & Wan L.-J. Nanostructured Materials for Electrochemical Energy Conversion and Storage Devices. Adv. Mater 20, 2878–2887 (2008).

[b7] Wu X.-L., Jiang L.-Y., Cao F.-F., Guo Y.-G. & Wan L.-J. LiFePO_4_ Nanoparticles Embedded in a Nanoporous Carbon Matrix: Superior Cathode Material for Electrochemical Energy-Storage Devices. Adv. Mater 21, 2710–2714 (2009). 10.1002/adma.200802998 36751060

[b8] Zhu C. B., Yu Y., Gu L., Weichert K. & Maier J. Electrospinning of Highly Electroactive Carbon-Coated Single-Crystalline LiFePO_4_ Nanowires. Angew. Chem., Int. Ed 50, 6278–6282 (2011). 10.1002/anie.201005428 21626615

[b9] Ke F.-S. . Fabrication and Properties of Three-Dimensional Macroporous Sn-Ni Alloy Electrodes of High Preferential (110) Orientation for Lithium Ion Batteries. Electrochem. Commun 9, 228–232 (2007).

[b10] Ji H.-X. . Self-wound Composite Nanomembranes as Electrode Materials for Lithium Ion. Adv. Mater 22, 4591–4595 (2010). 20839244 10.1002/adma.201001422

[b11] Lou X. W., Li C. M. & Archer L. A. Designed Synthesis of Coaxial SnO_2_@carbon Hollow Nanospheres for Highly Reversible Lithium Storage. Adv. Mater 21, 2536–2539 (2009).

[b12] Choi N. S. . Challenges Facing Lithium Batteries and Electrical Double-Layer Capacitors. Angew. Chem., Int. Ed 51, 9994–10024 (2012). 10.1002/anie.201201429 22965900

[b13] Bruce P. G., Scrosati B. & Tarascon J. M. Nanomaterials for Rechargeable Lithium Batteries. Angew. Chem. Int. Ed 47, 2930–2946 (2008). 10.1002/anie.200702505 18338357

[b14] Dai J. X., Li F. Y., Gao Z. Q. & Siow K. S. Low-Temperature Synthesized LiV_3_O_8_ as a Cathode Material for Rechargeable Lithium Batteries. J. Electrochem. Soc 145, 3057–3062 (1998).

[b15] West K. . Comparison of LiV_3_O_8_ Cathode Materials Prepared by Different Methods. J. Electrochem. Soc 143, 820–825 (1996).

[b16] Xu H. Y. . Novel Chemical Method for Synthesis of LiV_3_O_8_ Nanorods as Cathode Materials for Lithium Ion Batteries. Electrochim. Acta 49, 349–353 (2004).

[b17] Zhao M., Jiao L. F., Yuan H. T., Feng Y. & Zhang M. Study on the Silicon Doped Lithium Trivanadate as Cathode Material for Rechargeable Lithium Batteries. Solid State Ionics 178, 387–391 (2007).

[b18] Kawakita J., Kato T., Katayama Y., Miura T. & Kishi T. Lithium Insertion Behaviour of Li_1+x_V_3_O_8_ with Different Degrees of Crystallinity. J. Power Sources 81–82, 448–453 (1999).

[b19] Dubarry M., Gaubicher J., Guyomard D., Steunou N. & Livage J. Li_1+α_V_3_O_8_ Gel and Xerogel: A New Insight. Chem. Mater 16, 4867–4869 (2004).

[b20] Shi Q., Hu R. Z., Zeng M. Q. & Zhu M. A Diffusion Kinetics Study of Li-Ion in LiV_3_O_8_ Thin Film Electrode. Electrochim. Acta 55, 6645–6650 (2010).

[b21] Yang G., Wang G. & Hou W. H. Microwave Solid-State Synthesis of LiV_3_O_8_ as Cathode Material for Lithium Batteries. J. Phys. Chem. B 109, 11186–11196 (2005). 16852365 10.1021/jp050448s

[b22] Shi Q., Hu R. Z., Ouyang L. Z., Zeng M. Q. & Zhu M. High-Capacity LiV_3_O_8_ Thin-Film Cathode with a Mixed Amorphous-Nanocrystalline Microstructure Prepared by RF Magnetron Sputtering. Electrochem. Commun 11, 2169–2172 (2009).

[b23] Xiong X. H. . High Performance LiV_3_O_8_ Cathode Materials Prepared by Spray-Drying Method. Electrochim. Acta 71, 206–212 (2012).

[b24] Pan A. Q. . Template Free Synthesis of LiV_3_O_8_ Nanorods as a Cathode Material for High-Rate Secondary Lithium Batteries. J. Mater. Chem 21, 1153–1161 (2011).

[b25] Qiao Y. Q. . Synthesis and Electrochemical Performance of Rod-Like LiV_3_O_8_ Cathode Materials for Rechargeable Lithium Batteries. J. Power Sources 198, 287–293 (2012).

[b26] Pan A. Q. . Nanosheet-Structured LiV_3_O_8_ With High Capacity and Excellent Stability for High Energy Lithium Batteries. J. Mater. Chem 21, 10077–10084 (2011).

[b27] Wang R. S. . LiV_3_O_8_ Nanorods as Cathode Materials for High-Power and Long-Life Rechargeable Lithium-Ion Batteries. RSC Adv 4, 25494–25501 (2014).

[b28] Liang S. Q. . Facile Synthesis of Multiwalled Carbon Nanotube-LiV_3_O_8_ Nanocomposites as Cathode Materials for Li-Ion Batteries. Materials Letters 93, 435–438 (2013).

[b29] Idris N. H., Rahman M. M., Wang J. Z., Chen Z. X. & Liu H. K. Synthesis and Electrochemical Performance of LiV_3_O_8_/Carbon Nanosheet Composite as Cathode Material for Lithium-Ion Batteries. Composites Science and Technology 71, 343–349 (2011).

[b30] Li D. & Kaner R. B. Graphene-Based Materials. Science 320, 1170–1171 (2008). 18511678 10.1126/science.1158180

[b31] Li D., Muller M. B., Gilje S., Kaner R. B. & Wallace G. G. Processable Aqueous Dispersions of Graphene Nanosheets. Nat. Nanotechnol 3, 101–105 (2008). 18654470 10.1038/nnano.2007.451

[b32] Huang X., Qi X., Boey F. & Zhang H. Graphene-Based Composites. Chem. Soc. Rev 41, 666–686 (2012). 21796314 10.1039/c1cs15078b

[b33] Bai H., Li C. & Shi G. Q. Functional Composite Materials Based on Chemically Converted Graphene. Adv. Mater 23, 1089–1115 (2011). 21360763 10.1002/adma.201003753

[b34] Novoselov K. S. . Electric Field Effect in Atomically Thin Carbon Films. Science 306, 666–669 (2004). 15499015 10.1126/science.1102896

[b35] Geim A. K. & Novoselov K. S. The Rise of Graphene. Nat. Mater 6, 183–191 (2007). 17330084 10.1038/nmat1849

[b36] Wang H., Liang Y., Li Y. & Dai H. Co_1-x_S-Graphene Hybrid: A High-Performance Metal Chalcogenide Electrocatalyst for Oxygen Reduction. Angew. Chem., Int. Ed 50, 10969–10972 (2011). 10.1002/anie.201104004 21954126

[b37] Wang H., Robinson J. T., Diankov G. & Dai H. Nanocrystal Growth on Graphene with Various Degrees of Oxidation. J. Am. Chem. Soc 132, 3270–3271 (2010). 20166667 10.1021/ja100329d

[b38] Luo B. . Two Dimensional Graphene-SnS_2_ Hybrids with Superior Rate Capability for Lithium Ion Storage. Energy Environ. Sci 5, 5226–5230 (2012).

[b39] Loh K. P., Bao Q., Eda G. & Chhowalla M. Graphene Oxide as A Chemically Tunable Platform for Optical Applications. Nat. Chem 2, 1015–1024 (2010). 21107364 10.1038/nchem.907

[b40] Liang Y. Y., Li Y. G., Wang H. L. & Dai H. J. Strongly Coupled Inorganic/Nanocarbon Hybrid Materials for Advanced Electrocatalysis. J. Am. Chem. Soc 135, 2013–2036 (2013). 23339685 10.1021/ja3089923

[b41] Armand M. & Tarascon J.-M. Building Better Batteries. Nature 451, 652–657. (2008). 18256660 10.1038/451652a

[b42] Ye J. F. . Nanoporous Anatase TiO_2_ Mesocrystals: Additive-Free Synthesis, Remarkable Crystalline-Phase Stability, And Improved Lithium Insertion Behavior. J. Am. Chem. Soc 133, 933–940 (2011). 21142068 10.1021/ja108205q

[b43] Hu Y. S. . Improved Electrode Performance of Porous LiFePO_4_ Using RuO_2_ as An Oxidic Nanoscale Interconnect. Adv. Mater 19, 1963–1966 (2007).

[b44] Wang G. X., Shen X. P., Yao J. & Park J. Graphene Nanosheets for Enhanced Lithium Storage in Lithium Ion Batteries. Carbon 47, 2049–2053 (2009).

[b45] Ferrari A. C. & Robertson J. Interpretation of Raman Spectra of Disordered and Amorphous Carbon. Phys. Rev. B 61, 14095–14107 (2000).

[b46] Stankovich S. . Synthesis of Graphene-Based Nanosheets via Chemical Reduction of Exfoliated Graphite Oxide. Carbon 45, 1558–1565 (2007).

[b47] Zhong C., Wang J. Z., Chen Z. X. & Liu H. K. SnO_2_-Graphene Composite Synthesized via An Ultrafast and Environmentally Friendly Microwave Autoclave Method and Its Use as A Superior Anode for Lithium-Ion Batteries. J. Phy. Chem. C 115, 25115–25120 (2011).

[b48] Yoo E. . Large Reversible Li Storage of Graphene Nanosheet Families for Use in Rechargeable Lithium Ion Batteries. Nano Lett 8, 2277–2282 (2008). 18651781 10.1021/nl800957b

[b49] Wu Z. S. . Graphene/Metal Oxide Composite Electrode Materials for Energy Storage. Nano Energy 1, 107–131 (2012).

[b50] Wang H. L. & Dai J. H. Strongly Coupled Inorganic-Nano-Carbon Hybrid Materials for Energy Storage. Chem. Soc. Rev 42, 3088–3113 (2013). 23361617 10.1039/c2cs35307e

[b51] Yang S. . Graphene-Based Nanosheets with A Sandwich Structure. Angew. Chem., Int. Ed 49, 4795–4799 (2010). 10.1002/anie.201001634 20512835

[b52] Chen Y., Zhang X., Yu P. & Ma Y. W. Stable Dispersions of Graphene and Highly Conducting Graphene Films: A New Approach to Creating Colloids of Graphene Monolayers. Chem. Commun 20, 4527–4529 (2009). 10.1039/b907723e 19617972

[b53] Xu C. H., Sun J. & Gao L. Synthesis of Novel Hierarchical Graphene/ Polypyrrole Nanosheet Composites and Their Superior Electrochemical Performance. J. Mater. Chem 21, 11253–11258 (2011).

