# Supplementary material for: Ultradispersed Nanoarchitecture of LiV3O8 Nanoparticle/Reduced Graphene Oxide with High-Capacity and Long-Life Lithium-Ion Battery Cathodes
Source: Sci Rep. 2016 Jan 28;6:19843. doi: 10.1038/srep19843 (PMC4730191; doi:10.1038/srep19843)
Supplement: Supplementary Information [file srep19843-s1.doc]

Electronic Supplementary Information (ESI)

**Ultradispersed Nanoarchitecture of LiV_3_O_8_ Nanoparticle/ Reduced Graphene Oxide with High-Capacity and Long-Life Lithium-Ion Battery Cathodes**

By *Runwei Mo*^1,2^*, Ying Du*^1^*, David Rooney*^3^*, Guqiao Ding**^2^ *and Kening Sun**^1^

[1] Academy of Fundamental and Interdisciplinary Sciences, Harbin Institute of Technology, Harbin 150001, (China)

E-mail: [keningsunhit@126.com](mailto:keningsunhit@126.com)

[2] State Key Laboratory of Functional Materials for Informatics, Shanghai Institute of Microsystem and Information Technology, Shanghai, 20050, (China)

E-mail: gqding@mail.sim.ac.cn

[3] School of Chemistry and Chemical Engineering, Queen's University Belfast, Belfast, BT9 5AG, (Northern Ireland)


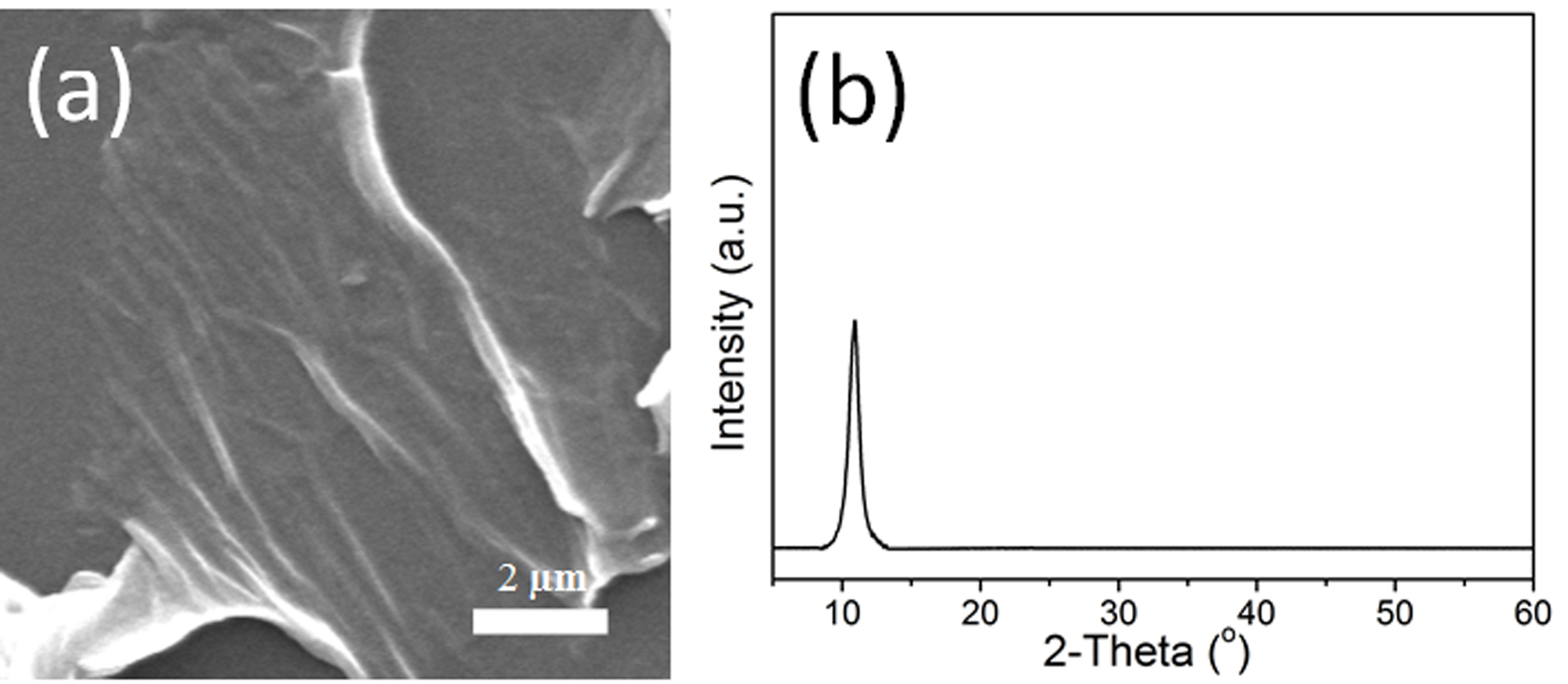


**Figure S1.** (a) SEM image of the GO. (b) XRD pattern of the GO.


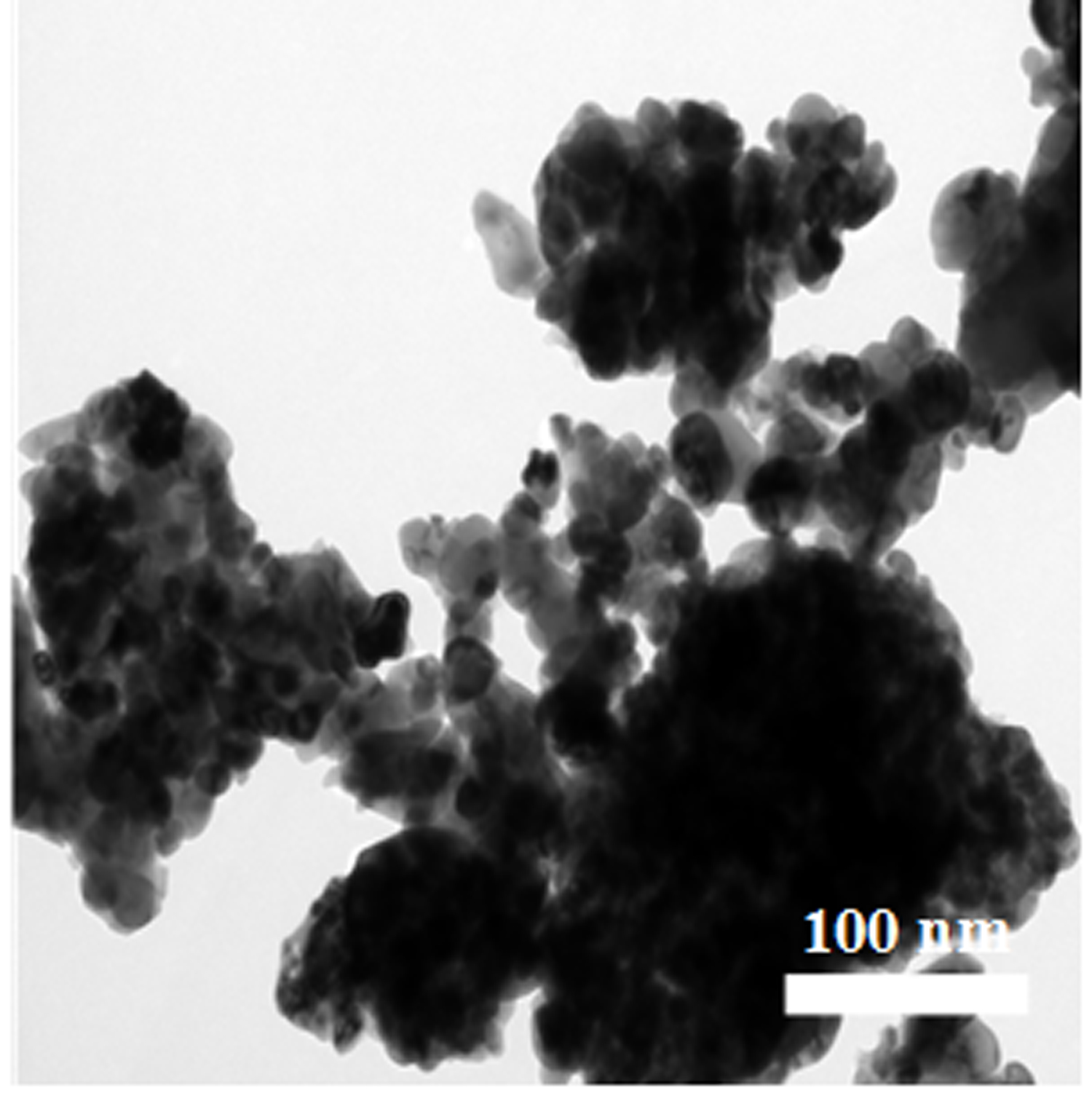


**Figure S2.** TEM images of LVO NPs synthesized without the addition of GNs.


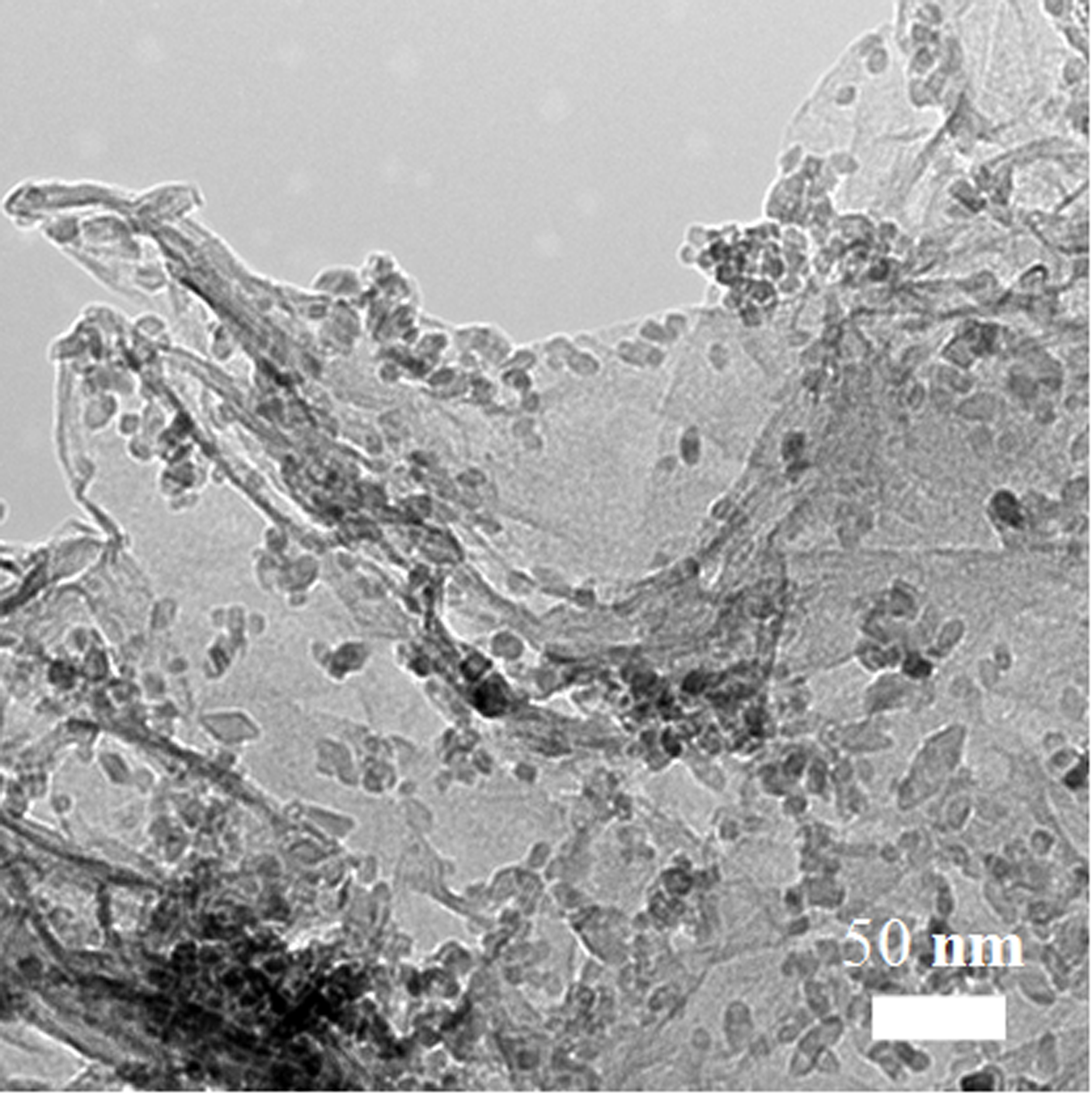


**Figure S3.** TEM images of the SG-LVO-GNs composites.


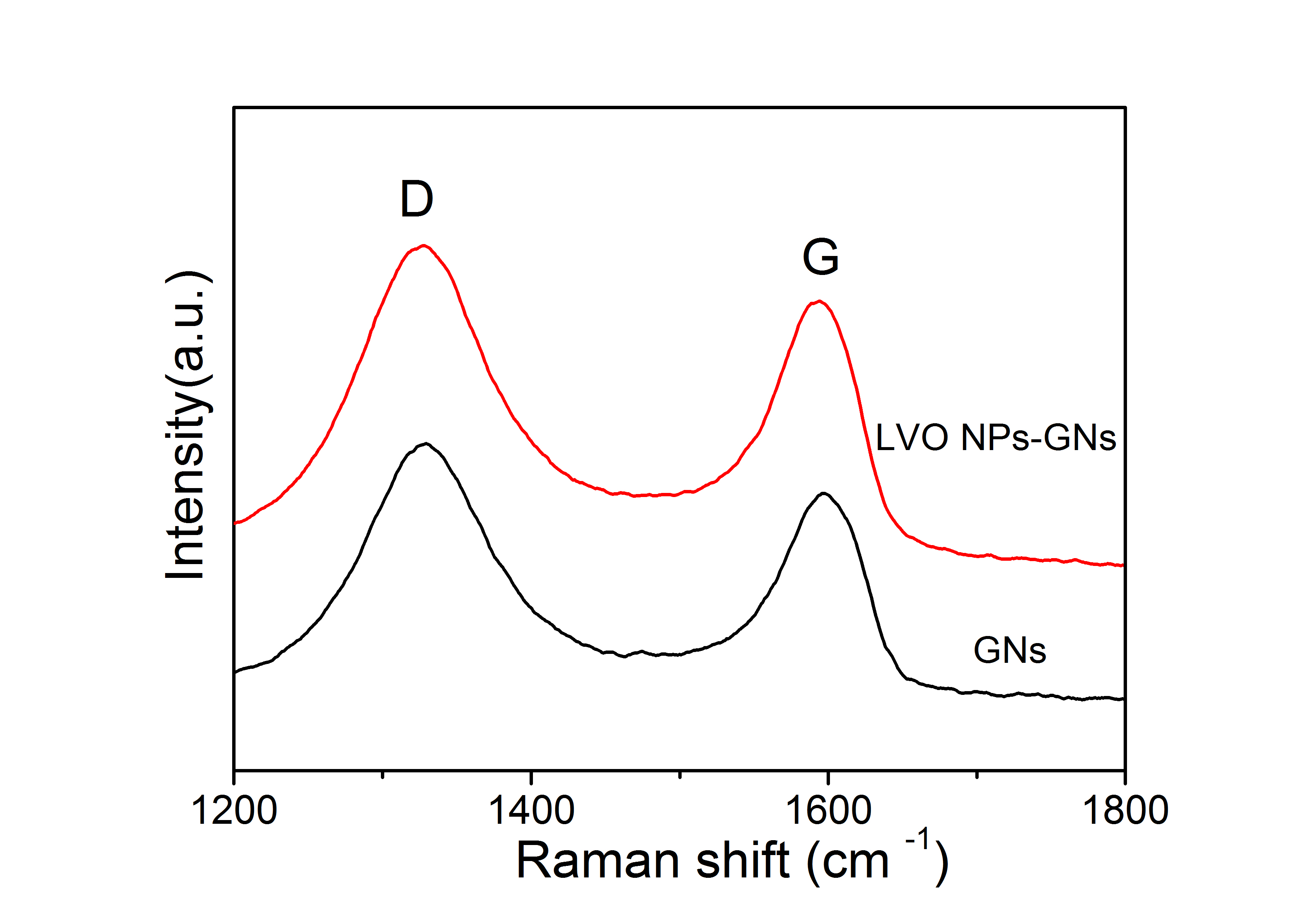


**Figure S4.** Raman spectra of LVO NCs-GNs and GNs in the wave length range of 1200-1800 cm^-1^.


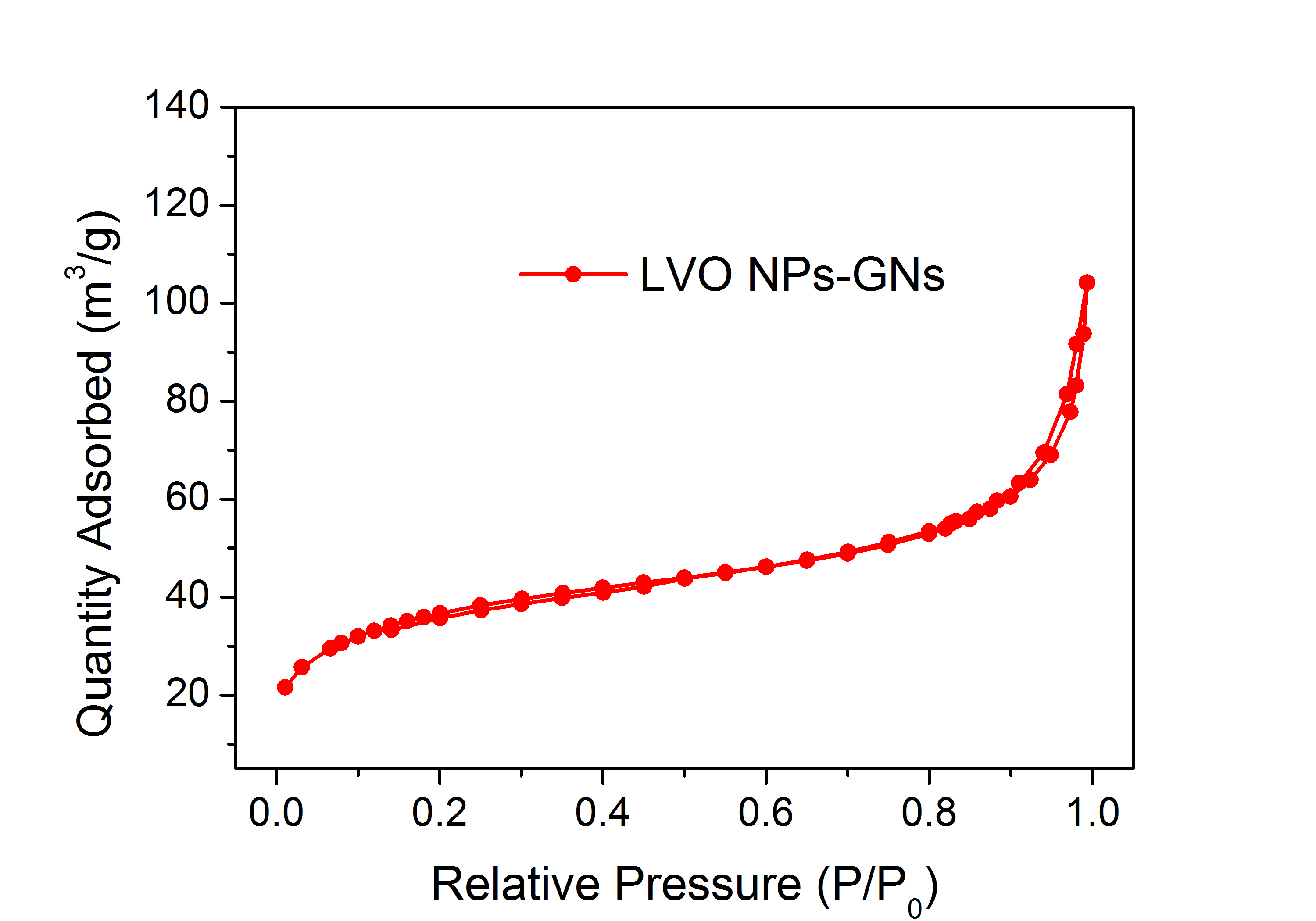


**Figure S5.** Nitrogen adsorption/desorption isotherms of the LVO NPs-GNs nanocomposite.


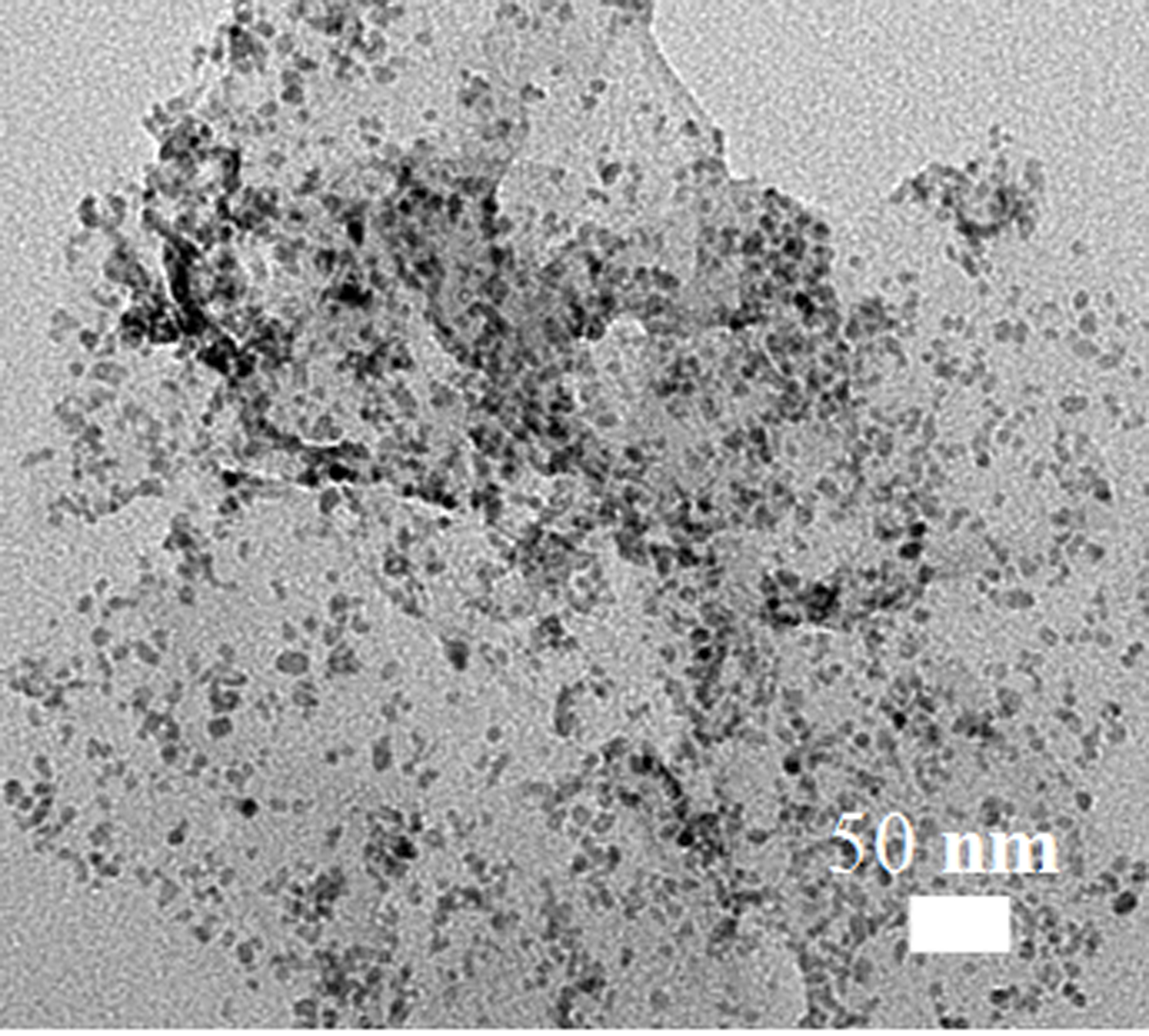


**Figure S6.** TEM image of LVO NCs-GNs after 200 discharge/charge cycles at current densities of 6 A g^-1^.
